# Effect of sex-specific differences on function of induced hepatocyte-like cells generated from male and female mouse embryonic fibroblasts

**DOI:** 10.1186/s13287-020-02100-z

**Published:** 2021-01-25

**Authors:** Imran Ullah, Yurianna Shin, Yeongji Kim, Keon Bong Oh, Seongsoo Hwang, Young-Im Kim, Jeong Woong Lee, Tai-Young Hur, Seunghoon Lee, Sun A Ock

**Affiliations:** 1 Animal Biotechnology Division, National Institute of Animal Science, Rural Development Administration, 1500 Kongjwipatjwi-ro, Isero-myeon, Wanju-gun, Jeollabuk-do 565-851 Republic of Korea; 2 Biotherapeutics Translational Research Center, Korea Research Institute of Bioscience and Biotechnology, 125, Gwakhak-ro, Yuseong-gu, Daejeon, 34141 Republic of Korea; 3 Department of Biochemistry, Faculty of Biological Sciences, Quaid-i-Azam University, Islamabad, Pakistan

**Keywords:** Mouse embryonic fibroblasts, Induced hepatocytes, Sex specific, Hormone, Liver

## Abstract

**Background:**

The liver is one of the vital organs involved in detoxification and metabolism. The sex-based differences between the functionality of male and female liver have been previously reported, i.e., male’s liver are good in alcohol clearance and lipid metabolism, while female’s liver are better in cholesterol metabolism. To date, studies on novel drug toxicity have not considered the sex-specific dimorphic nature of the liver. However, the use of hepatocyte-like cells to treat liver diseases has increased recently.

**Methods:**

Mouse embryos were isolated from a pregnant female C57BL/6J mouse where mouse embryonic fibroblasts (MEFs) were isolated from back skin tissue of each embryo. MEFs were transduced with human transcription factors hHnf1α, hHnf4α, and hFoxa3 using the lentiviral system. The transduced MEFs were further treated with hepatocyte-conditioned media followed by its analysis through RT-qPCR, immunofluorescence, functional assays, and finally whole-transcriptome RNA sequencing analysis. For in vivo investigation, the mouse hepatocyte-like cells (miHep) were transplanted into CCl4-induced acute liver mouse model.

**Results:**

In this study, we evaluated the sex-specific effect of miHep induced from male- and female-specific mouse embryonic fibroblasts (MEFs). We observed miHeps with a polygonal cytoplasm and bipolar nucleus and found that male miHeps showed higher *mHnf4a*, albumin secretion, and polyploidization than female miHeps. Transcriptomes from miHeps were similar to those from the liver, especially for *Hnf4a* of male miHeps. Male *Cyps* were normalized to those from females, which revealed *Cyp* expression differences between liver and miHeps. In both liver and miHeps, *Cyp 4a12a* and *Cyp 4b13a/2b9* predominated in males and females, respectively. After grafting of miHeps, AST/ALT decreased, regardless of mouse sex.

**Conclusion:**

In conclusion, activation of endogenic *Hnf4a* is important for generation of successful sex-specific miHeps; furthermore, the male-derived miHep exhibits comparatively enhanced hepatic features than those of female miHep.

## Background

The liver is a vital organ for metabolism, synthesis, and detoxification. In addition to performing multiple similar functions in both males and females, the liver also exhibits sex-based differences. Males show a high liver to weight ratio and excellent alcohol clearance and lipid metabolism ability [1, 2], while in females, cholesterol metabolism ability is enhanced [3, 4]. Various factors such as aging, accidents, and liver diseases can cause liver damage. Liver transplantation is the optimal solution for liver damage achieving a survival rate of 50% in some acute liver failure patients [5]. However, due to the growing demand for liver transplantation and an increasing rate of liver diseases, such as hepatitis A, C, and cirrhosis, liver organ donations are insufficient to meet the demand [6].

Hepatocyte transplantation is considered an alternative treatment for metabolic liver disorders or liver failure caused by liver cancer and cirrhosis. Only a small fraction of the liver is replaced during hepatocyte transplantation [7]. The first hepatocyte transplantation was attempted in the rat portal vein [8] and was then successfully performed in humans through the splenic arterial infusion technique. However, with current methods, hepatocytes obtained from donor livers are of lower quality, fewer in number, and difficult to expand in vitro and cryopreserve [9]. Moreover, hepatocytes cannot maintain their metabolic function in vitro under long-term culturing conditions. Therefore, stem cell therapy was introduced as a substitute for the generation of hepatocytes. Stem cells have the potential to differentiate into specialized cell types such as beta cells, neuron-like cells, cardiomyocytes, and hepatocytes [10]. Recently, direct conversion technology using somatic cells has been deciphered, where mouse fibroblasts can be directly converted into specific hepatocyte-like cells [11, 12]. Such functional induced hepatocyte-like cells (iHeps) were generated using combination of defined transcription factors, Gata4/Foxa3/Hnf1a, Hnf4a/Foxa1, 2, or 3, HNF1A/HNF4A/HNF6/ATF5/PROX1/CEBPA [11–14], and small molecules, A83-01 and CHIR99021 [15]. These in vitro reprogrammed stem cells have been successfully used in vivo in animal models with liver diseases [13, 15, 16].

As a “sexually dimorphic organ,” the liver is affected by gonad hormones such as androgen and estrogen and displays “drug toxicity and drug-dose sex gap” [17]. However, the combined effect of sex chromosomes and gonad hormones on iHep function has not been examined. Therefore, here, we evaluated the effect of sex chromosomes on the function of mouse iHeps (miHeps) generated with hepatic transcription factors (hHnf1α, hHnf4α, hFoxa3) and small molecules (A-83-01) in vitro. Furthermore, male and female miHeps were transplanted into liver failure animal models, and the effect of gonad hormones were tested using serum biochemical analyses to examine liver function.

## Materials and methods

### Reagents and media

Unless otherwise specified, all chemicals were purchased from Sigma-Aldrich Corporation (St. Louis, MO, USA), and media were obtained from Gibco (Invitrogen, Burlington, ON, Canada).

### Experimental animals used for analysis of liver function

C57BL/6J mice (11-week-old) were obtained from Japan SLC (Shizuoka, Japan). Livers were isolated from five male and five female mice from the same litter. All experiments were performed under the guidelines of the Institutional Animal Care and Use Committee of the National Institute of Animal Science.

### Isolation and culture of mouse embryonic fibroblasts (MEFs)

Mouse embryos [embryonic day 13 (E13)] were isolated from a pregnant female C57BL/6J mouse (Japan SLC, Shizuoka, Japan). The back skin tissue of each embryo was collected and treated with collagenase to obtain single isolated MEF. The isolated MEFs were cultured in Advanced Dulbecco’s modified Eagle’s medium (A-DMEM) containing 10% fetal bovine serum (FBS; Gibco 10082147), 1× penicillin/streptomycin, and 1× glutamax. Cells were sub-cultured until passages 3–6 and were then used for further studies.

### Polymerase chain reaction (PCR) analysis for sex-determination

Genomic DNA was extracted from the MEFs (passage 3) using the DNeasy blood and tissue kit (Qiagen, Hilden, Germany) and amplified using Prime Taq Premix (2×) (GenetBio). A 50 ng DNA sample was used for PCR with the following conditions: for *Dax-1*, denaturation at 94 °C for 5 min, followed by 35 cycles of annealing and extension at 94 °C for 30 s, 57 °C for 30 s, and 72 °C for 30 s; for *Sry* and *Gapdh*, denaturation at 94 °C for 5 min, followed by 32 cycles of annealing and extension at 94 °C for 30 s, 55 °C for 30 s, and 72 °C for 30 s (Supplementary Table 1). The PCR products were subjected to agarose electrophoresis.

### Lentivirus production

Lentiviruses were produced through transfections in 293T cells (Clontech, Mountain View CA USA) with Lipofectamine 3000 reagent (Invitrogen), using a previously reported modified method [11, 16]. The human transcription factors HNF1A, HNF4A, and FOXA3 were cloned together into the dsRed plasmid site.

### Generation of miHeps

A total of 5 × 10^4^ MEF cells were seeded on collagen-coated 35-mm dishes and were cultured in A-DMEM supplemented with 1 μM 5-Azacytidine and 10% FBS. After 24 h, cells were infected with lentivirus vectors containing three human transcription factors (HNF1A, HNF4A, and FOXA3) at a multiplicity of infection (MOI) of 1 and were cultured in infection medium (A-DMEM supplemented with 3% FBS). Two days after infection, the culture medium was replaced with hepatocyte induction medium (HIM) [William’s E medium supplemented with 5% FBS, 1× penicillin/streptomycin, 1× glutamax, 0.1 mg/mL ornithine, 0.61 mg/mL nicotinamide, 1× ITS, 40 ng/mL TGFα, 40 ng/mL EGF, 10 ng/mL HGF, 10 μM dexamethasone (DEX), and 10 ng/mL oncostatin M] along with 2 μM A-83-01 for maintaining hepatocyte function and morphology.

### Gene expression analysis by quantitative real-time PCR

Total RNA was isolated using an RNeasy mini kit (Qiagen, Hilden, Germany). cDNA was synthesized from total purified RNA (500 ng) with Omniscript RT kit (Qiagen, Hilden, Germany) according to the manufacturer’s instructions. Real-time PCR was performed using a StepOnePlus Real-Time PCR system (Applied Biosystem, CA, USA) with SYBR green PCR master Mix (Thermo Fisher Scientific, CA, USA) using 10 μM of a specific primer set (Supplementary Table 2). *Gapdh* was used as the internal control gene and all samples were run in 5 replicates.

### Immunocytofluorescence staining of hepatocyte-specific proteins

For confirmation of hepatocyte-specific protein expression, cells were fixed with 3.7% formalin for 30 min at room temperature (RT) and then blocked in Dulbecco’s phosphate-buffered saline (DPBS) containing 3% BSA and 0.1% Triton X-100 (BioRad) for 20 min at RT. Cells were incubated with primary antibodies (Supplementary Table 1) at 4 °C overnight. After primary antibody treatment, cells were washed twice with DPBS containing 3% BSA and incubated with appropriate secondary antibodies (Supplementary Table 3) for 1 h at RT in the dark. For nuclear staining, cells were stained with 1 μg/mL DAPI solution for 30 min at RT, washed twice with DPBS containing 3% BSA, and mounted with Vectashield (Vector Laboratories, Inc., Burlingame, CA., USA). Finally, cells were observed under a fluorescence microscope (Leica DMI 6000B, Germany).

### In vitro hepatocyte function assay

For detection of neutral triglycerides/lipids and glycogen accumulation, cells were fixed with 3.7% formalin for 30 min and washed twice with DPBS. The cells were then stained with 3 mg/mL Oil Red O solution for 30 min or subjected to periodic acid-Schiff (PAS, Millipore, Billerica MC, USA) staining at RT, according to the manufacturer’s instructions. Post-staining, the cells were washed several times with distilled water. In case of PAS staining, counter staining was performed with Mayer’s hematoxylin for 1 min. The samples were observed under a light microscope.

For the assay measuring cellular uptake and release of indocyanine green (ICG), healthy cells were incubated with 1 mg/mL ICG solution for 1 h at 37 °C and washed twice with DPBS before ICG uptake was observed under a light microscope. They were then incubated in HIM without ICG for 6 h at 37 °C, after which ICG release was observed under a microscope.

For the acetylated low-density lipoprotein (ac-LDL) uptake assay, healthy cells were incubated with 10 μg/mL Dil-Ac-LDL (ac-LDL labeled with 1,1′-dioctadecyl-3,3,3′-tetramethylindo-carbocyanine perchlorate) (Invitrogen) for 3 h at 37 °C, counter stained with DAPI, and then evaluated for red signal (indicative of a positive reaction) under a fluorescence microscope.

### ELISA to measure albumin and urea secretion

To measure the amount of mouse albumin and urea in culture media, cell culture supernatants from miHeps were collected on days 2 and 3 (day 0 = seeding day). HIM was used as a control. Albumin and urea levels were determined using the Mouse Albumin Enzyme-Linked Immunosorbent Assay (ELISA) kit (Abcam, Cambridge, MA, UK) and Urea Assay kit (Abcam, Cambridge, MA, UK), respectively, and readings were taken with the iMark™ Microplate Absorbance Reader (BIO-RAD, Hercules, California, USA) with Microplate Manager software 6 (BIO-RAD, Hercules, California, USA). Each experiment was performed in triplicate (Supplementary Fig. 1).

### CYP activity test using specific chemical inducers

To assess expression levels of CYP enzymes, miHeps were cultured in HIM supplemented with 10 μM 3-methylcholanthrene (3-MC, Sigma, St. Louis, Mo.) [18, 19], 25 μM rifampicin (RIF, Sigma, St. Louis, Mo.) [15, 16], and 100 μM DEX (Sigma, St. Louis, Mo.) [15] for 72 h at 37 °C. After induction, total RNA was extracted and CYP genes respondent to the inducer were analyzed by real-time PCR using a specific set of primers (Supplementary Table 2).

### Karyotyping and ploidy analysis of miHep using flow cytometry

At passage 9–10, 30 cells per group for both male and female miHeps were analyzed for chromosome structure and karyotyping using the GTG Banding method by a chromosome analysis company (Korea Research of Animal Chromosomes, Seoul, Korea). Meanwhile, cells from the same passage were fixed with cold 70% ethanol, washed twice with PBS, and stained with 1 μg/mL propidium iodide (PI), before DNA content was confirmed through cell cycle analysis using flow cytometry. The original MEF of each group was used as the control. DNA content was classified, as previously reported [20].

### Whole-transcriptome RNA sequencing analysis

For whole-transcriptome RNA sequencing analysis, 1 μg total RNA was extracted from each sample of male and female MEFs (negative control), male and female miHeps, and mouse liver (positive control). RNA sequencing libraries were prepared with Agilent 2100 BioAnalyzer High Sensitivity DNA kit (Agilent. Technologies, Waldbronn, Germany). The libraries were sequenced using the HiSeq 2000 sequencing system (San Diego, CA USA). Data were normalized to MEFs, miHeps, or female liver tissue.

### In vivo test with miHeps and serum biochemical analysis

For the in vivo test, we used 40 BALB/c Cr Slc -nu/nu nude mice (6–7 weeks old; Japan SLC, Shizuoka, Japan). The animals were divided into group of five where the first five animals (control mice) were not given any carbon tetrachloride (CCl_4_, Sigma, MO, USA) treatment. Further, five animals were sacrificed at day 1 and at day 4 post-CCl4 treatment, respectively (Supplementary Figure 7). Furthermore, five male and five female mice were transplanted with male iHeps at day 1 post-CCl_4_ treatment followed by five male and five female animals transplanted with female iHeps at day 1 post-CCl_4_ treatment. Finally, five male animals were injected with PBS at day 1 post-CCl_4_ treatment. For induction of acute liver injury, CCl_4_ was injected into the abdominal cavity of mice using a method reported by Lim et al. [15]. Briefly, 6 to 7-week-old BALB/c Cr Slc -nu/nu nude mice received an intraperitoneal injection of 25% CCl4 (100 μl/20 g body weight) in corn oil. After 1 day, 1.5–2 × 10^6^ miHeps/100 μL PBS stained with pkh26 (Sigma-Aldrich, MO, USA) were surgically transplanted into the spleen of mice under anesthesia. After 3 days, all mice were sacrificed, and total blood was harvested for serum biochemical analysis [aspartate aminotransferase (AST), alanine aminotransferase (ALT), alkaline phosphatase (ALP), albumin (ALB*)*, total bilirubin (T-BIL), and total protein (TP)] using the Hitachi clinical analyzer 7180 (Hitachi. Ltd., Tokyo, Japan). The liver was also harvested to confirm localization of transplanted miHeps. All animal tests and care were approved by the Institutional Animal Care and Use Committee of the NIAS. Harvested livers were fixed with 3.7% formalin, washed twice with PBS, and used to prepare paraffin blocks or OCT Compound for further experiments. The prepared blocks were subjected to immunohistochemistry analyses by hematoxylin/eosin (H&E) staining or Masson’s trichrome staining for detection of collagen fiber and immunofluorescence staining to detect pkh26*.* Counter-staining was performed with DAPI. All slide samples were observed by light or fluorescence microscopy.

### Statistical analysis

For statistical analysis, data from at least three replicates were used and analyzed with a one-way ANOVA and post hoc Tukey or LSD test using IBM SPSS statistics software, version 24. PCR data are expressed as relative quantification (RQ), and error bars represent the RQ ± min and max RQ values. Other data are represented as mean ± standard deviation (SD). A *p* value < 0.05 was considered statistically significant.

## Results

### Analysis of hepatocyte function-related major genes in mouse livers

**Fig. 1:**
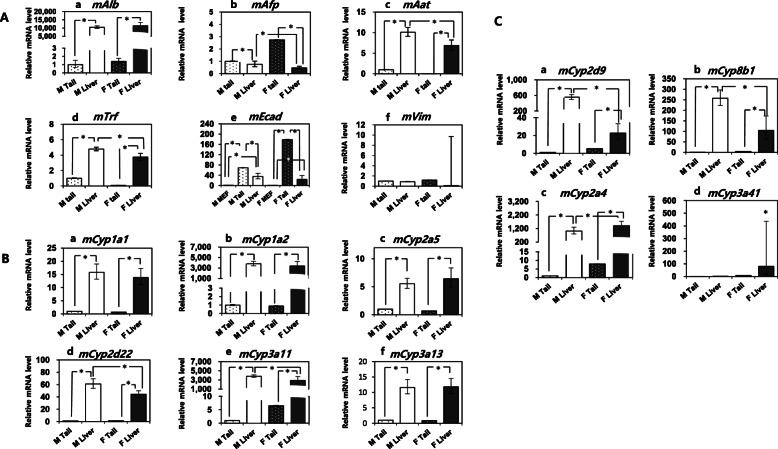
Analysis of hepatocyte-specific genes and CYP genes in mouse livers. Real-time PCR analysis of (**A**) hepatocyte-specific genes, (**B**) major hepatocyte-specific Cyp genes, and (**C**) sex-dependent Cyp genes in male and female mouse livers. Expression levels were normalized to those of the male mouse tail. Generation of miHeps derived from MEFs. Five biological replicates were performed (5 male and 5 female mice). *Above the bars represent a statistically significant difference at *p* < 0.05. The data represent the relative quantification (RQ) ± min and max RQ values

**Fig. 2:**
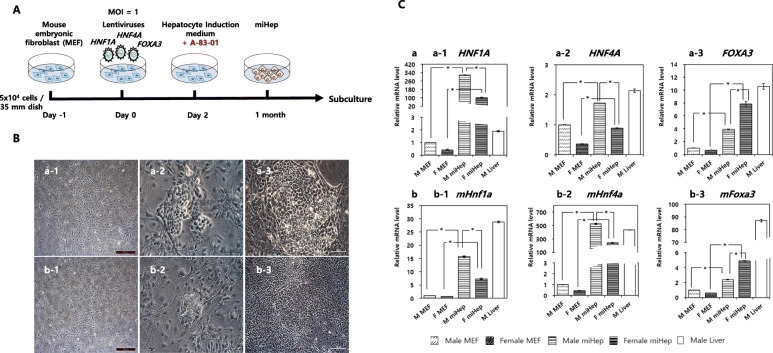
Activation of external and internal transcriptional factors in induced miHeps. (**A**) Direct differentiation strategy for miHep generation. (**B**) Morphological changes during miHep generation, (a-1) male MEF and (b-1) female MEF upon initial seeding. After 1 month of induction, (a-2) male and (b-2) female MEFs successfully differentiated into male and female miHeps, respectively. The number of colonies increased with passage of time, and (a-3; b-3) both miHeps showed bipolar cells and polygonal morphology with various sizes of colonies. (**C**) The expression of (Ca) exogenic and (Cb) endogenic transcription factor genes in induced male and female miHeps. Male liver tissue were used as a negative control (Ca) or positive control (Cb) referenced to transcription factors. *Above the bars represent a statistically significant difference at *p* < 0.05. The data represent the relative quantification (RQ) ± min and max RQ values

Genes related to hepatocyte function were investigated in male and female mouse livers to identify sex-dependent characteristics (Fig. 2 and Supplementary Fig. 1). All gene expression levels were normalized using male mouse tail tissue, except for the epithelial cadherin gene (*Ecad*), which was normalized using male MEF. The *Alb*, *Aat*, and *Trf* genes, which are involved in protein synthesis in mature hepatocytes, were expressed in all mice, regardless of sex. *Aat* and *Trf* showed significantly higher expression in male livers than in female livers, while *Alb* did not show any sex-based differences in expression (Fig. 1A). Expression of *AfP*, a gene involved in protein production in fetal liver, was decreased in both male (0.43-fold) and female (0.30-fold) mice (Fig. 1Ab). *Ecad* expression was increased in both male (34.94-fold) and female (23.68-fold) livers, but was not significantly different among different sexes. Vim, a mesenchymal cell marker (type III intermediate filament protein), did not increase in either male or female livers.

Furthermore, the expression patterns of major CYP genes, such as *Cyp1a1*, *Cyp1a2*, *Cyp2a5*, and *Cyp3a13*, were similar in both males and females (Fig. 1B); however, *Cyp2d22* and *Cyp3a11* expression was significantly higher in the male than in the female liver. *Cyp* genes encoding for sex-dependent CYP enzymes, such as *Cyp2d9* and *Cyp8b1*, were more prevalent in the male liver than in the female liver, although the reverse was true for *Cyp2a4* and *Cyp3a41* (Fig. 1C)*.* Among individual mice, expression levels of the abovementioned genes showed slight differences (Supplementary Fig. 2).

Based on the results shown in Figure S1, male and female MEFs were randomly selected for generation of miHeps. After transfection of viral vectors, the formation of miHeps was observed at 2 weeks (Fig. 2). The miHeps exhibited colonies of varying sizes and had a bipolar nucleus and polygonal cytoplasm after 4 weeks, regardless of the sex (Fig. 2B). Colonies were manually picked under a stereo microscope for sub-culturing, up to passage 3. From passage 4 onwards, cells were divided at 1:4 ratio for subsequent passages.

### Expression of exo/endogenic hepatic transcription factors

Exo/endogenous hepatic transcription factors were analyzed by real-time PCR 3 months after transfection of viral vectors (Fig. 2C). All exogenic hepatocyte transcription factors were upregulated in both male and female miHeps, compared with their sex-matched MEFs, especially *HNF1Α* for male miHeps and *FOXA3* for female miHeps (Fig. 2Ca). The sex-based expression patterns of each endogenic hepatocyte transcription factor were similar to that of exogenic hepatocyte transcription factors, although the absolute values of each gene differed (Fig. 2Cb). Particularly, *mHnf4α* (male miHep, 526.21-fold; female miHep, 241.27-fold) was expressed at a higher level than the other genes (Fig. 2Cb-2). Furthermore, all mouse hepatocyte transcription genes were upregulated in the mouse liver, though each was upregulated to a different degree.

### Expression of hepatocyte-specific genes and proteins

**Fig. 3:**
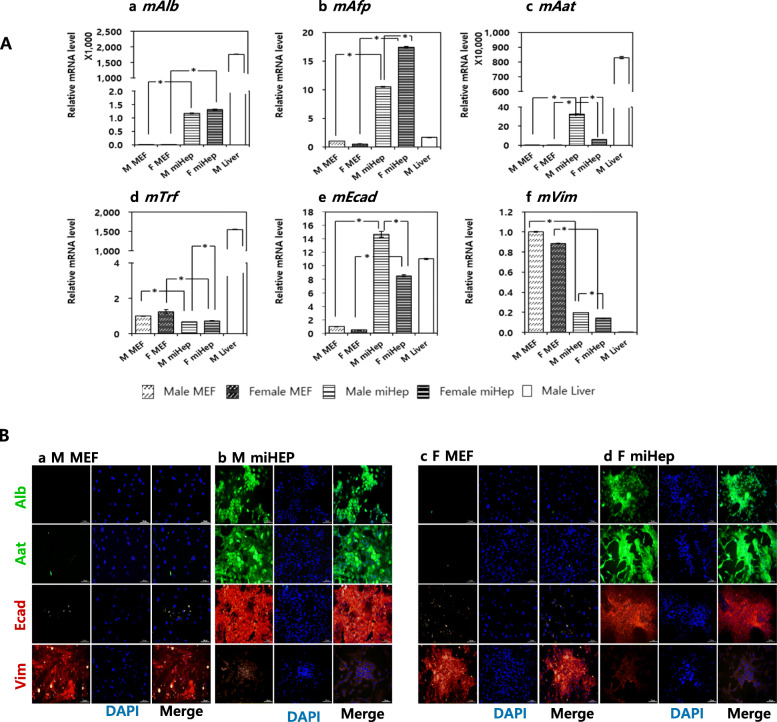
Expression of hepatocyte-specific genes and proteins in miHep. (**A**) Hepatocyte-specific markers, (a) *mAlb*, (b) *mAfp*, (c) *mAat*, (d) *mTrf*, (e) *mEcad*, and (f) *mVim* analyzed by real-time quantitative PCR. MEFs and liver were used as a negative and positive control for each gene, respectively. *Above the bars represent a statistically significant difference at *p* < 0.05. Male liver tissue was used as a positive control for hepatocyte-specific genes. (**B**) Comparison of expression of hepatocyte-specific proteins (Alb, Aat, and Ecad) between (Bb) male and (Bd) female miHeps. MEFs were used as a negative control for each protein. Green and red fluorescence indicate positive expression of each protein, and blue fluorescence indicates DAPI staining of the nucleus. Scale bar = 100 μm. The data represent the relative quantification (RQ) ± min and max RQ values

Next, we analyzed the expression of major hepatocyte-specific genes and proteins (Fig. 3). The expression of *mAlb*, *mAat*, and *mAfp*, which are involved in the synthesis of major proteins in hepatocytes, increased in both male and female miHeps. Between male and female miHeps, *mAlb* showed the same expression level (male, 1170.24-fold; female, 1307.89-fold; Fig. 3Aa), *mAat* predominated in the males (male, 324,261.52-fold; female, 60,595.36-fold, Fig. 3Ac), while *mAfp* was predominant in the females (male, 10.50-fold; female, 17.43-fold, Fig. 3Ab). Other major hepatocyte-specific genes, such as *mTrf*, were not expressed in both male and female miHeps (Fig. 3Ad). *mEcad*, an epithelial cell marker, was increased in both male and female miHeps, and especially in male miHeps (Fig. 3Ae). In contrast, *mVim*, a mesenchymal cell marker, was dramatically decreased in both male and female miHeps (Fig. 3Af).

An analysis of the corresponding proteins was performed in MEFs and miHeps (Fig. 3B). Regardless of sex, MEF, as a negative control, did not express Alb, Aat, and Ecad proteins, but strongly expressed the mesenchymal marker Vim. In contrast, this expression pattern was reversed in miHeps.

### Expression of major xenobiotic metabolizing enzymes and activity of Cyp enzymes upon treatment with specific Cyp inducers

**Fig. 4:**
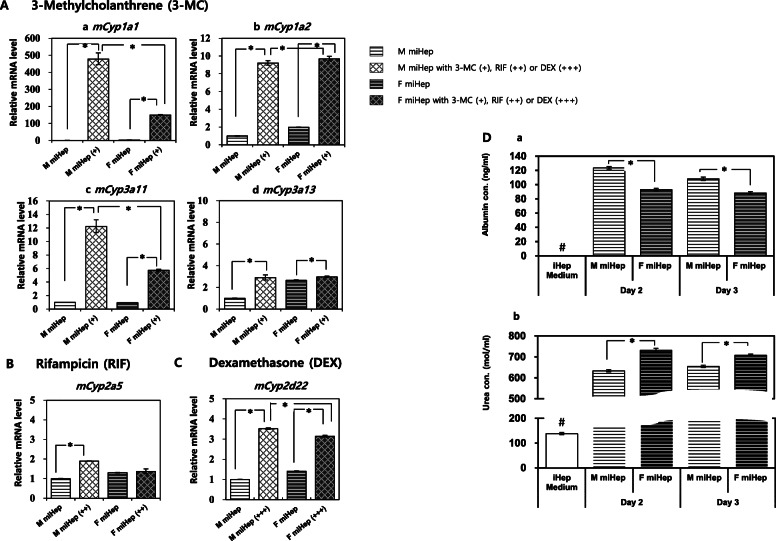
Xenobiotic metabolizing Cyps analysis and albumin and urea secretion in miHep. miHeps treated for 3 days with specific Cyp inducers, 3-methylcholanthrene (3-MC, +) for *Cyp1a1 *(a), *Cyp 1a2* (b), *Cyp 3a11 *(c) and *Cyp 3a13* (d); Rifampicin (RIF, ++) for *Cyp 2a5* (**B**); and dexamethasone (DEX, +++) for *Cyp 2d22* (**C**). Relative mRNA levels were normalized to male miHeps, in the absence of chemicals. The data represent the relative quantification (RQ) ± min and max RQ values. *Above the bars represent a statistically significant difference at *p* < 0.05. (**D**) Secretion levels of (a) albumin and (b) urea by ELISA in each cell culture supernatant of male and female miHeps cultured for 2 and 3 days. Fresh culture medium for miHeps was used as control. *Above the bars represent a statistically significant difference at *p* < 0.05. # indicates statistically significant difference with all groups. The data represent means ± SD

Expression of the major CYP enzymes in miHeps was analyzed both at the gene and protein levels (Supplementary Fig. 4). *Cyp*s increased in both male and female miHeps, except for two genes, m*Cyp1a2* and *mCyp2a5* (Supplementary Fig. 4Ab, c), although their expression levels compared to the positive control were low. In miHeps, the expression pattern of *Cyps* was sex-dependent, since most *Cyps* (1a1, 2a5, 2d22, and 3a11) were more prevalent in female miHeps than in male miHeps (Fig. 4Aa–e), except for *mCyp3a13* (Supplementary Fig. 4Af).

Furthermore, the proteins Cyp3a11 (red fluorescence) and Cyp1a2 (green fluorescence) were positively expressed in the cytoplasm of both male and female miHeps (Supplementary Fig. 4B). Moreover, Cyp3a11 was more strongly expressed than Cyp1a2, regardless of sex (Supplementary Fig. 4B).

Regardless of sex specificity, 3-MC treatment induced the upregulation of *mCyp1a1, mCyp1a2, mCyp3a11*, and *mCyp3a13*, particularly *mCyp1a1* (male, 478.37-fold; female, 149.61-fold, Fig. 4A), where *mCyp1a1* and *mCyp3a11* expression was more predominant in male miHeps. RIF treatment upregulated *mCyp2a5* expression slightly in male, but not in female miHeps (without RIF, 1.30-fold; with RIF, 1.37-fold) (Fig. 4B). DEX treatment induced upregulation of *mCyp2d22* in both male and female miHeps (Fig. 4C), especially in the male miHeps (without DEX, 1.00-fold; with DEX, 3.52-fold).

### Analysis of physiological functions of hepatocytes

The physiological functions of hepatocytes were evaluated in the miHeps via histochemical staining for neutral lipids (Supplementary Fig. 5Aa), glycogen storage (Supplementary Fig. 1Ab), ac-LDL intake (Supplementary Fig. 5Ac), and ICG absorption and release (Supplementary Fig. 5Ad). Results of all histochemical staining showed a positive reaction in both male and female miHeps. In case of oil red O staining, several large red droplets were observed in male miHeps, compared to female miHeps. In PAS staining, female miHeps showed more predominant staining than the male miHeps, while for Ac-LDL and ICG uptake/release, the results were the opposite. *mLpl* and *mPparγ*, which are involved in lipid metabolism, were upregulated in male compared to female miHeps, which is consistent with the superior capacity of the male miHeps to accumulate lipid compared with female miHeps (Supplementary Fig. 5B).

Next, secretion of albumin and urea into miHep culture supernatant was confirmed for both male and female miHeps using ELISA (Fig. 4D). Albumin secretion was higher in males, while urea secretion was higher in female miHeps, regardless of culturing time.

### Polyploidization of miHeps

**Fig. 5:**
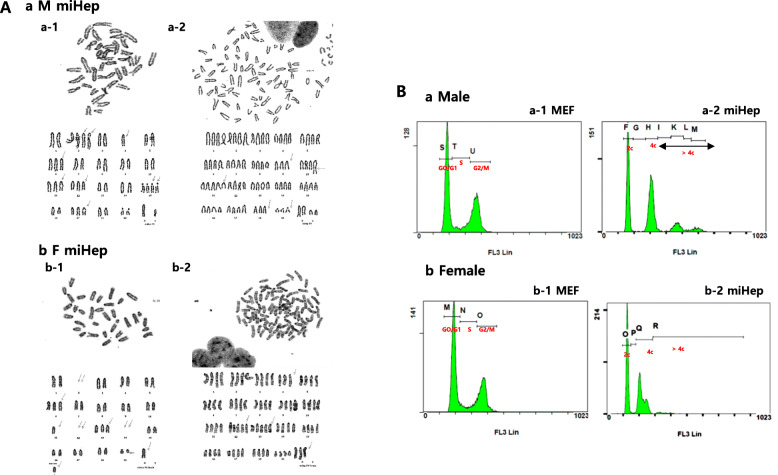
Karyotyping and polyploidy analysis of miHeps. (**A**) The plotted karyotypes of (a) male and (b) female miHeps, respectively. a-1 and b-1 closed to ~ 2n, aneuploidy, and a-2 and b-2 closed to ~ 4n. (**B**) Analysis of cell cycle and ploidy by flow cytometry after PI staining in MEFs and miHeps. (a-2) Male and (b-2) female miHeps originated from (a-1) male MEFs and (b-1) female MEFs used for this analysis, respectively

The chromosome status of miHeps was evaluated via karyotyping and flow cytometry analysis (Fig. 5). Both male and female miHeps showed frequent polyploidy, including 3n~4n, in addition to aneuploidy, as seen through karyotyping, especially in male miHeps (Fig. 5A). To confirm the ploidy of miHeps, we carried out flow cytometry analysis. Both male and female MEFs used for induction of miHeps demonstrated a normal cell cycle (Fig. 5Ba-1/b-1) and normal ploidy and karyotype (Supplementary Fig. 3B). However, male miHeps showed 4 clear peaks, while female miHeps showed 2 clear peaks, with the second peak being the highest. The first and second peaks of each MEF were shifted to the left in both miHeps, regardless of sex. The presence of aneuploidy supported variation in DNA content (Supplementary Table 4). Ratios above ~ 4C were 21% in male miHeps and 6.6% in female miHeps. Together, our results indicate that polyploidy and aneuploidy are phenomena predominant in male miHeps, rather than in female miHeps.

### Analysis of whole-transcriptome RNA sequencing data

**Fig. 6:**
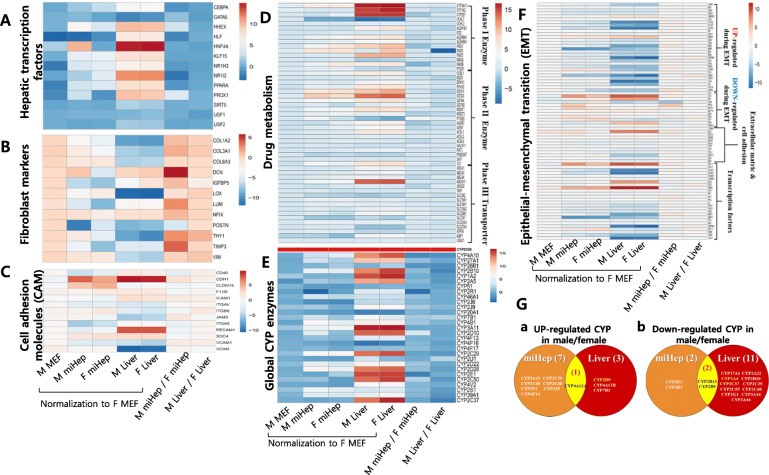
Global gene expression of miHep by whole transcriptome analysis. The heat maps represent (**A**) hepatocyte transcription factor genes, (**B**) fibroblast marker genes, (**C**) cell adhesion molecules (CAM)-related genes, (**D**) drug metabolism related gene including phase I, II enzyme and 3 transporter genes, (**E**) global CYP enzymes, and (**F**) epithelial–mesenchymal transition (EMT) pathway-related genes, respectively. Male MEF, male miHep, female miHep, male liver and female liver normalized with female MEF. male miHep / female miHep meant male miHep normalized with female miHep. Male liver / female liver meant male liver normalized with female liver. (**G**) Venn diagram of up (a) or down (b) regulated Cyp genes between male miHeps and male liver normalized with female male miHeps and liver, respectively

Genes encoding hepatic transcription factors showed strong upregulation in both male and female livers with only minor differences (Fig. 6A). Of these, *Hnf4a* was most strongly expressed in the liver. Overall, gene expression in male and female miHeps was similar to that seen for the liver, especially *Hnf4a* expression in male miHeps. Fibroblast marker showed reduced expression in the liver as compared to MEFs, and both male and female miHeps also were downregulated, though not to the extent found in the liver (Fig. 6B). Cell adhesion molecule genes (CAM) did not exhibit sex-based differences in the liver, and *Cdh1* (*E-cad*) and *Pecam1* showed higher expression than other liver genes. Both miHeps, especially male miHeps, had similar expression patterns in the liver (Fig. 6C). The expression of genes *Fmo*3, *Gsta1, Gsta1*, and *Abcb11*, which encode drug metabolism enzymes, including phase I/II and phase III enzymes, increased only slightly in female livers, compared to that in male livers. The overall expression pattern for genes in male and female miHeps was similar to that in sex-matched livers, although the expression degrees varied (Fig. 6D). Global *Cyp* enzymes were more predominant in female than in male livers, while the opposite trend was observed for miHeps (Fig. 6E). Expression of epithelial-mesenchymal transition (EMT)-related genes was altered in the liver, such that upregulated genes were suppressed and downregulated genes were induced. Similar patterns were found in both male and female miHeps, and this was especially true for male miHeps (Fig. 6F). Furthermore, glucose and lipoprotein/cholesterol metabolism activities were slightly higher in female miHeps (Supplementary Fig. 6A, B), while fatty acid metabolism was upregulated in male miHeps (Supplementary Fig. 6C).

To compare sex-dependent CYP gene expression levels in liver and miHeps, a 2-fold change was designated as the cut-off (Fig. 6G). In males, liver or miHep expression was normalized to that of the female liver or miHeps, respectively. The number of upregulated genes was 4 (*2d9, 4a12a, 4a12b, 7b1*) in the liver and 8 (*2a12, 2c50, 2c65, 2c68, 2f2, 2j5, 4a12a, 4f14*) in miHeps. Only one gene was co-upregulated in the male liver and miHeps, namely *Cyp4a12a*. The number of downregulated genes in the male liver and miHeps was 13 (*2a4, 2a22, 3a16, 3a44, 2b10, 2b9, 2b13, 2c37, 2c39, 2c55, 2c68, 2 g1*) and 4 genes (*2b13, 2b9*, *2e1, 2r1*), respectively, and the number of co-downregulated genes was 2, namely *Cyp2b9* and *Cyp2b13*. Overall, females showed stronger CYP gene expression than males in the liver, but not in miHeps, respectively.

### In vivo effect of miHep transplantation in an acute liver injury model

**Fig. 7:**
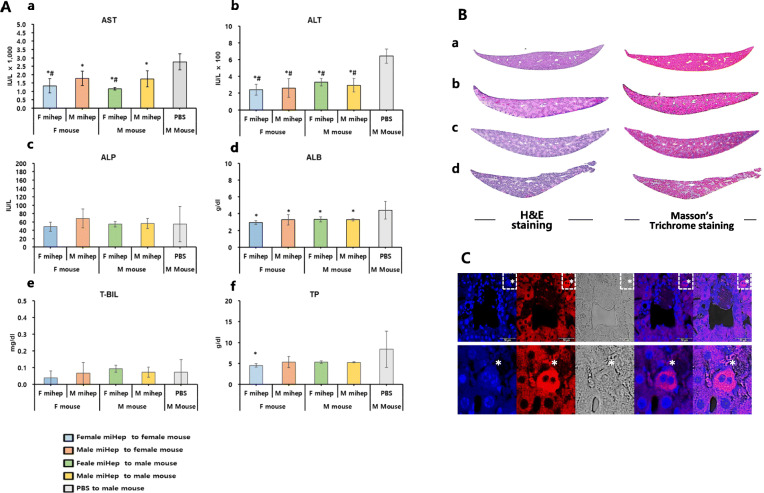
Liver function in acute liver injury model transplanted with miHep. (**A**) Serum of mice was collected 4 days after CCl4 injection and analyzed for aspartate aminotransferase (AST, a), alanine aminotransferase (ALT, b), alkaline phosphatase (ALP, c), albumin (ALB, d), total bilirubin (T-BIL, e), and total protein (TP, f) using a Hitachi clinical analyzer 7180 (Hitachi. Ltd., Tokyo, Japan). Before transplantation, 1–2 × 10^6^ miHeps labeled with Pkh26 (Red fluorescence) and transfused into spleen of female and male mouse (5 mice/group). PBS group used as manipulated control (*n* = 5). * or # above the bars represent a statistically significant difference by LSD or Tukey HSD post hoc test, respectively at *p* < 0.05. The data represent means ± SD. (**B**) Section of liver transplanted with miHep stained with hematoxylin/eosin (H&E) or Masson’s trichrome staining for detection of collagen fiber. A health liver; b, liver at 1 day after CCl4 injection; c, liver at 3 days after transfusion of PBS; d liver at 3 days after transfusion of miHep. (**C**) Section of liver transplanted with male miHep stained by Pkh26. Red signals found around blood vessel. Blue (DAPI) signals meant counter staining to confirm nucleus of cell. White dot squares enlarged as shown below. White asterisks indicate cells with a bipolar nucleus originated from male miHep

Immunodeficient mice 1 day post-CCl_4_ treatment showed severe centrilobular necrosis and hemorrhage (Fig. 7Bb) by H&E staining. Moreover, a weak blue stain around liver lobules was observed by Masson’s trichrome staining (Fig. 7Bb). Serum from mice showed a dramatic increase in AST (12,948.33 IU/L), ALT (13,088 IU/L), and T-BIL (0.16 mg/dl) levels, and a decline in ALP (80.67 IU/L) by the LSD post hoc test (Supplementary Fig. 7). Four days after CCl_4_ injection, levels of AST, ALT, and T-BIL were reduced, although not to control levels. Thus, we created an acute liver injury model with CCl_4_ treatment for use in cell transplantation experiments.

miHeps were grafted into the acute liver injury model mouse (Fig. 7 Bd). Histochemical analysis did not reveal differences between PBS (control) and miHep transplanted groups. However, serum biochemical analysis results revealed some differences. The miHep groups showed significantly decreased levels of AST, ALT, and ALB compared with the PBS-treated group, as determined through the LSD post hoc test, regardless of sex matching between miHep and mice. However, when a Tukey HSD post hoc test was applied, only mice transplanted with female miHeps showed a statistically significant decline in these enzymes, and this was especially prominent for male (*p* = 0.005) compared with female (*p* = 0.011) mice. ALP and T-BIL levels were not affected by grafting of miHeps compared with PBS. The miHeps transplanted into the acute liver injury model were observed under a fluorescence microscope after cryosection of liver tissue, and pkh26-labeled hepatocytes were found with two nuclei around the vessel (Fig. 7C). Together, our results show that sex matching between donor miHeps and recipient mice does not affect recovery of liver function.

## Discussion

In this study, we evaluated whether miHeps generated from male or female MEFs could reflect the physiological characteristics of sex-dependent hepatocytes through both in vitro and in vivo experiments. We found that male and female miHeps reflected the basic characteristics of sex-dependent hepatocytes in vitro, but were not affected by short-term exposure of gonad hormones in vivo. Moreover, activation of major transcription genes used for miHep generation contributed to the unique function of mature hepatocytes.

We successfully generated miHeps from male or female MEFs with a Lentiviral vector system, including hepatic transcription factors (*HNF1A, HNF4A, FOXA3*). Transfection efficiency of three exogenic hepatic transcription factors affected the expression of three endogenic hepatic transcription factors in both male and female miHeps. We observed a dramatic upregulation of endogenic mouse Hnf4a, similar to levels seen in the mouse liver. Hnf4a is reportedly important for differentiation, glycogen storage, and epithelium generation in hepatocytes [21]. Further, it controls endoderm formation from embryonic stem cells and hepatic progenitor cells via induction and maintenance of multiple hepatic transcription factors, including Fox3 and Hnf1a [22]. Therefore, endogenic Hnf4a overexpression in male miHeps induced the ability to differentiate into functional hepatocytes.

The miHeps we generated exhibit shared characteristics with the adult liver, as is evident from expression of *Alb, Aat, Ecad,* and *Vim* genes, which have functions and physiological characteristics similar to mature hepatocytes, such as lipid/glycogen accumulation, ac-LDL uptake, ICG uptake/release, and increase of drug metabolism-related CYP enzyme genes [23]. These findings were consistent with previous reports that evaluated the same hepatic transcription factors [16] in a different species. Furthermore, previous reports of miHeps treated with specific CYP inducers indicated a dramatic increase in expression of major CYP genes, such as *Cyp1a1/1a2*, *Cyp2a5,* and *Cyp3a11/3a13* [15, 24], consistent with our results. Thus, we confirmed generation of functional miHeps from male and female MEFs, and male miHep was superior to female miHep in terms of stable expression of *Afp/Ecad*, activation ability of major Cyp genes (*Cyp 1a1 *and *3a11*), upregulation of adipocytes genes (*Lpl/Pparγ*), and elevated albumin secretion.

Sex-specific effect on miHeps generated from male and female MEFs was assessed by whole transcriptome analysis via RNA sequencing. Compared with female miHeps, male miHeps had expression patterns more similar to the liver regarding the expression of hepatic transcriptional factors, such as Hnf4a, fibroblast-specific genes, and cell adhesion molecules (CAM), including *Cdh1* and *EMT*. In the male liver, the number of up- or downregulated CYP enzyme was 4 and 14, respectively, although male miHeps showed the opposite pattern (expression was normalized to female liver and miHep, respectively). The number of co-up or down regulated genes between the liver and miHep was 1 (*Cyp4a12a*, 2.47-fold) or 2 (*Cyp2b13*, − 3.33-fold / *2b9*, − 2.02-fold), respectively. These genes demonstrated a sex-based predominance pattern in the liver. Sex-dependent mouse Cyp genes showed a large difference in expression level (up to 500-fold) between males and females, with *Cyp 2a4, 2b9,12a1, 2b13* higher in females, and *Cyp4a12, 2d9, 7b1* dominant in males [25]. Male and female miHeps reflected this sex-related predominance in Cyp expression pattern, and we additionally found that activation of major transcription factors, such as endogenic *Hnf4a*, was important for the expression of Cyp genes. This finding was supported by an increase in upregulated Cyp gene number in male miHeps, as compared to the male liver.

Polyploidy in the liver has long been described as not only an age-dependent but also a species-dependent process [26–29]. Generally, the hepatocyte is diploid at birth and gives rise to production of binucleated hepatocytes due to failed cytokinesis during postnatal growth. Binucleated hepatocytes can serve as a source for the development of polyploid mononucleated cells [30, 31]. This hepatic polyploidy may also serve to enhance the functional capacity of the liver, where aneuploid hepatocytes play an important role in responding to chronic liver injury [27]. We confirmed the diversity of DNA content through flow cytometry and karyotyping in both male and female miHeps, particularly in male miHeps. In contrast to our study, a study examining human-induced hepatocytes generated with identical transcription factors maintained a diploid nature [16]; this difference may be attributed to species. Another group also reported normal diploid-induced hepatocytes in mice [15] and humans [13], although some transcription factors used for induction of hepatocyte-like cells were inconsistent and detailed ploidy was not verified through flow cytometric analysis. Therefore, we assume that the miHeps in our study possess characteristics similar to mature hepatocytes based on diversity of DNA content, especially male miHeps. In addition to revealing polyploidy in mice, previous studies also reported that ~ 90% of hepatocytes are polyploid in adult C57Bl mice and that ~ 60% of hepatocytes in mice show aneuploidy by flow cytometric analysis [20, 28, 30].

The liver is reportedly a sexually dimorphic organ possessing both androgen and estrogen receptor to respond to sex hormones [17, 32]. Therefore, we tested recovery of liver function when male and female miHeps were exposed to gonad hormones in an in vivo model. Regardless of sex, effectiveness of transplanted iHeps were proved by improvement of ALT and AST levels in mouse live acute injury model. Estrogen reduces reactive oxygen species (ROS), redox hepatocellular apoptosis, and hepatic stellate cell activation [17]. However, in our study, male and female miHeps were not affected by short-term exposure of gonad hormones. The CCl_4_ used for induction of acute liver injury can produce severe ROS due to xenobiotic decomposition mediated by Cyp2e1, leading to death of hepatocytes, resulting in high Cyp2e1 expression [33]. As a result, Cyp2e1 mRNA decreases in the acute liver injury model. We found that both Cyp2e1 and the major Cyps (1a1, 2a5, 2d22, 3a11) related to xenobiotic decomposition were increased in female miHeps compared to male miHeps. One explanation for this finding is that female miHeps may allow recovery of hepatocyte function (AST) by replenishment of deficient Cyp mRNA under stress environment. This phenomenon supported by sex-specific difference which responded to chronic and acute change was explained by the innate difference of X-ist of females and methylation pattern of CYP family which rely on sex [34]. However, further studies are needed to determine why female miHeps show more efficient reduction of AST and how miHeps will behave in long-term exposure to gonad hormones.

## Conclusion

Expandable miHeps reflecting sex-dependent major *Cyp* expression patterns were successfully generated from male and female MEFs by a direct conversion method using transcription factors with the addition of small molecules. We showed upregulation of *Hnf4a*, which contributed to the function of mature hepatocyte by downregulating *Afp* and upregulating *Cdh1*, albumin secretion, and EMT pathway inhibition. Moreover, we found that *Hnf4a* upregulation leads to upregulation of a number of Cyps as well as increase in polyploidy rate. Therefore, we hypothesize that miHeps generation efficiency is influenced by two main factors; sex-specific differences and external artificial manipulation, such as transfection efficiency of major transcription factors. Finally, this study will be useful to further investigate differences in drug metabolism based on sex differences.

## Supplementary Information

**Additional file 1: Supplementary Figure 1.** Analysis of albumin (A) and urea levels (B) by Mouse Albumin ELISA kit and Urea assay kit, respectively. Color changes of cell culture supernatants before reading of the absorbance by a microplate reader. Black and red boxes indicate serially diluted albumin or urea standards, and samples used for analysis, respectively. **Supplementary Figure 2.** Analysis of major liver function and Cyp enzyme-related genes in individual male and female mice. A, major liver function and mature related genes; B, major Cyp genes related to xenobiotic decomposition; C, sex predominant Cyp genes. Mail tail tissue was used as negative control. Five male and five female mice were used for each specific mRNA analysis. Each gene was normalized with tail mRNA of single male mouse1 randomly selected. **Supplementary Figure 3.** Sex determination of MEFs and karyotyping of random selected MEF. A, after isolation of MEF from each fetus, sex was identified by sorting sex chromosome genes using PCR analysis. Electrophoresis revealed 1, 2, and 5 MEF as male, and 3, 4, 6, and 7 as female. For further analysis, MEF 1 from male and MEF 4 from female were chosen randomly. B, karyotyping results of randomly chosen male (a) and female (b) MEFs showed a normal karyotype. **Supplementary Figure 4.** Expression analysis of the major CYP enzymes in miHeps. (A) The expression of the major Cyp enzymes, (a) m *Cyp1a1*, (b) m *Cyp1a2*, (c) *mCyp2a5*, (d) m *Cyp2d22*, (e) *mCyp3a11*, and (f) m *Cyp3a13* was analyzed by real-time quantitative PCR in male and female miHeps. MEF and liver were used as negative and positive controls for each gene, respectively. Letters above the bars represent a significant difference at *p* < 0.05. The data represents relative quantification (RQ) ± min and max RQ values. (B) Expression of Cyp3a11 (red) and Cyp1a2/1a1 (green) proteins by immunofluorescence staining. MEF was used as a negative control. Blue fluorescence indicates DAPI staining of the nucleus. Scale bar = 100 μm. **Supplementary Figure 5.** Functional analysis of miHeps. (A) Histochemical analysis of miHeps by (a) Oil Red O staining, (b) PAS staining, (c) Dil-Ac-LDL uptake and (d-1,2) ICG uptake and release assay. Scale bar = 100 μm. (B) Analysis of *mLpl* and *mPparγ* by real-time quantitative PCR analysis. Superscript (*) above the bars represent a significant difference at *p* < 0.05. The data represents relative quantification (RQ) ± min and max RQ values. **Supplementary Figure 6.** Glucose/glycogen, lipoprotein/cholesterol and fatty acid metabolism of miHep by whole transcriptome analysis. A, Glucose/glycogen metabolism; B, lipoprotein/cholesterol metabolism; C, and fatty acid metabolism. Male MEF, male miHep, female miHep, male liver and female liver normalized with female MEF. male miHep / female miHep indicates male miHep normalized with female miHep. Male liver / female liver indicates male liver normalized with female liver. **Supplementary Figure 7.** Generation of a mouse model of acute liver injury. CCl4 diluted with Con oil was injected into the peritoneal cavity of immunodeficient mice. Sera were recovered from mice day 1 (*n* = 5) and 4 days (*n* = 5) after CCl4 injection and subjected to serum biochemical analysis [aspartate aminotransferase (AST, A), alanine aminotransferase (ALT, B), alkaline phosphatase (ALP, C), albumin (ALB, D), total bilirubin (T-BIL, E) and total protein (TP, F)] using a Hitachi clinical analyzer 7180 (Hitachi. Ltd., Tokyo, Japan). The data represent the means ± SD. Superscript (*) above the bars represent a significant difference by LSD post-hoc test at *p* < 0.05.

**Additional file 2: Supplementary Table 1.** Primers used for realtime PCR. **Supplementary Table 2.** Primers used for real-time PCR analysis. **Supplementary Table 3.** List of antibodies used for immunofluorescence staining. **Supplementary Table 4.** Ploidy percentage of miHep.

## Data Availability

All data generated or analyzed during this study are included in this article along with its supplementary information files.

## Abbreviations

MEF: Mouse embryonic fibroblasts
iHeps: Induced hepatocyte-like cells
MOI: Multiplicity of infection
HIM: Hepatocyte induction medium
3-MC: 3-methylcholanthrene
RIF: Rifampicin
ac-LDL: Acetylated low-density lipoprotein
DEX: Dexamethasone
ELISA: Enzyme-Linked Immunosorbent Assay
ICG: Indocyanine green
CCl_4_: Carbon tetrachloride
H&E: Hematoxylin and eosin
AST: Aspartate aminotransferase
ALT: Alanine aminotransferase
ALP: A*lkaline phosphatase*
ALB: A*lbumin*
T-BIL: Total b*ilirubin*
TP: Total protein
CYP: Cytochrome P450
