# Supplementary material for: Effect of sex-specific differences on function of induced hepatocyte-like cells generated from male and female mouse embryonic fibroblasts
Source: Stem Cell Res Ther. 2021 Jan 25;12:79. doi: 10.1186/s13287-020-02100-z (PMC7831237; doi:10.1186/s13287-020-02100-z)
Supplement: Supplementary file 1 — Additional file 1: Supplementary Figure 1. Analysis of albumin (A) and urea levels (B) by Mouse Albumin ELISA kit and Urea assay kit, respectively. Color changes of cell culture supernatants before reading of the absorbance by a microplate reader. Black and red boxes indicate serially diluted albumin or urea standards, and samples used for analysis, respectively. Supplementary Figure 2. Analysis of major liver function and Cyp enzyme-related genes in individual male and female mice. A, major liver function and mature related genes; B, major Cyp genes related to xenobiotic decomposition; C, sex predominant Cyp genes. Mail tail tissue was used as negative control. Five male and five female mice were used for each specific mRNA analysis. Each gene was normalized with tail mRNA of single male mouse1 randomly selected. Supplementary Figure 3. Sex determination of MEFs and karyotyping of random selected MEF. A, after isolation of MEF from each fetus, sex was identified by sorting sex chromosome genes using PCR analysis. Electrophoresis revealed 1, 2, and 5 MEF as male, and 3, 4, 6, and 7 as female. For further analysis, MEF 1 from male and MEF 4 from female were chosen randomly. B, karyotyping results of randomly chosen male (a) and female (b) MEFs showed a normal karyotype. Supplementary Figure 4. Expression analysis of the major CYP enzymes in miHeps. (A) The expression of the major Cyp enzymes, (a) m Cyp1a1, (b) m Cyp1a2, (c) mCyp2a5, (d) m Cyp2d22, (e) mCyp3a11, and (f) m Cyp3a13 was analyzed by real-time quantitative PCR in male and female miHeps. MEF and liver were used as negative and positive controls for each gene, respectively. Letters above the bars represent a significant difference at p < 0.05. The data represents relative quantification (RQ) ± min and max RQ values. (B) Expression of Cyp3a11 (red) and Cyp1a2/1a1 (green) proteins by immunofluorescence staining. MEF was used as a negative control. Blue fluorescence indicates DAPI staining of the nucleus. Scale [file 13287_2020_2100_MOESM1_ESM.pptx]

## Slide 1
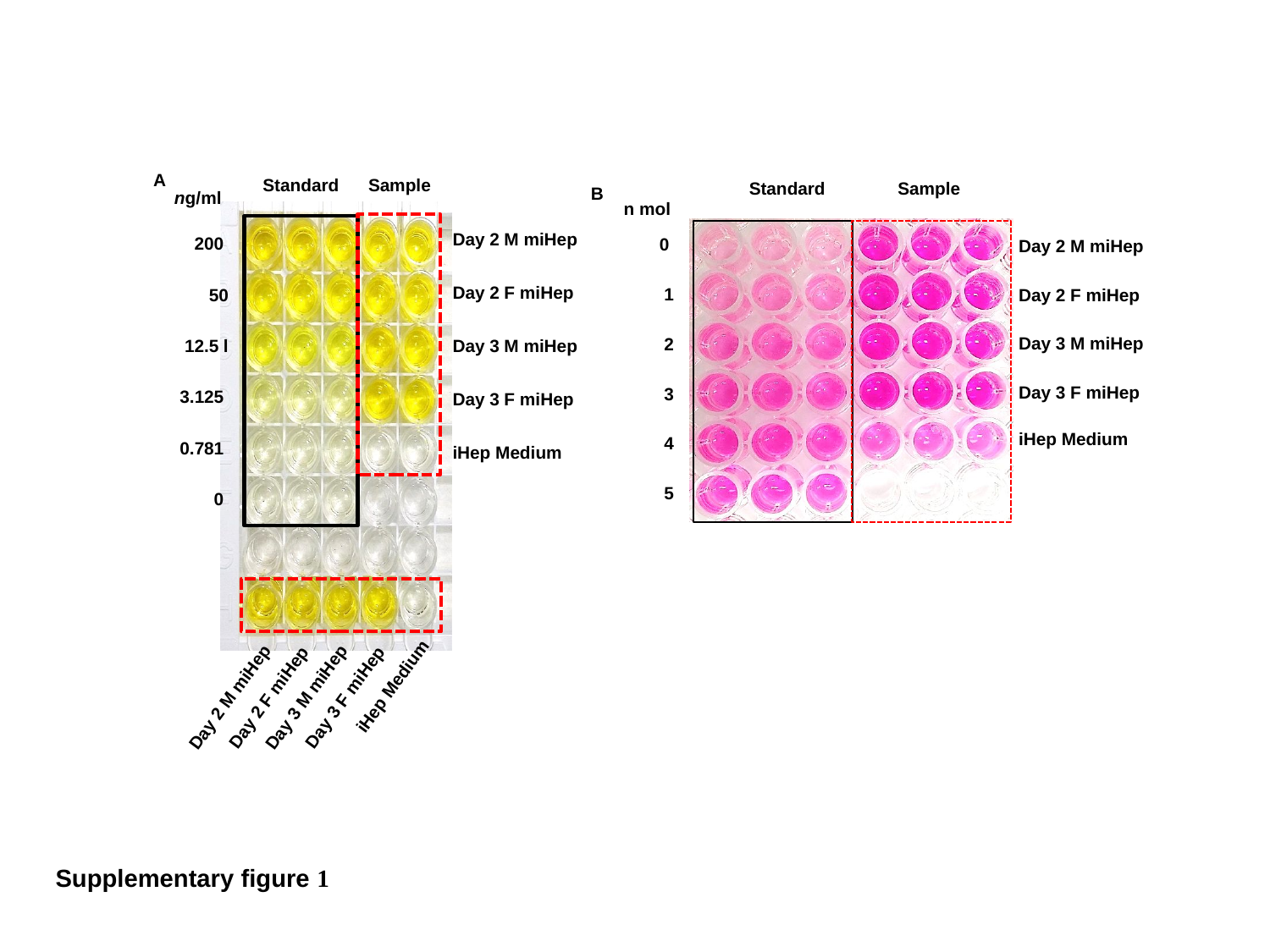

A
Standard
Sample
Standard
Sample
B
ng/ml
n mol
Day 2 M miHep
200
0
Day 2 M miHep
50
Day 2 F miHep
1
Day 2 F miHep
12.5 l
Day 3 M miHep
2
Day 3 M miHep
Day 3 F miHep
3
3.125
Day 3 F miHep
iHep Medium
4
0.781
iHep Medium
5
0
Day 2 M miHep
Day 2 F miHep
Day 3 M miHep
Day 3 F miHep
iHep Medium
Supplementary figure 1

## Slide 2
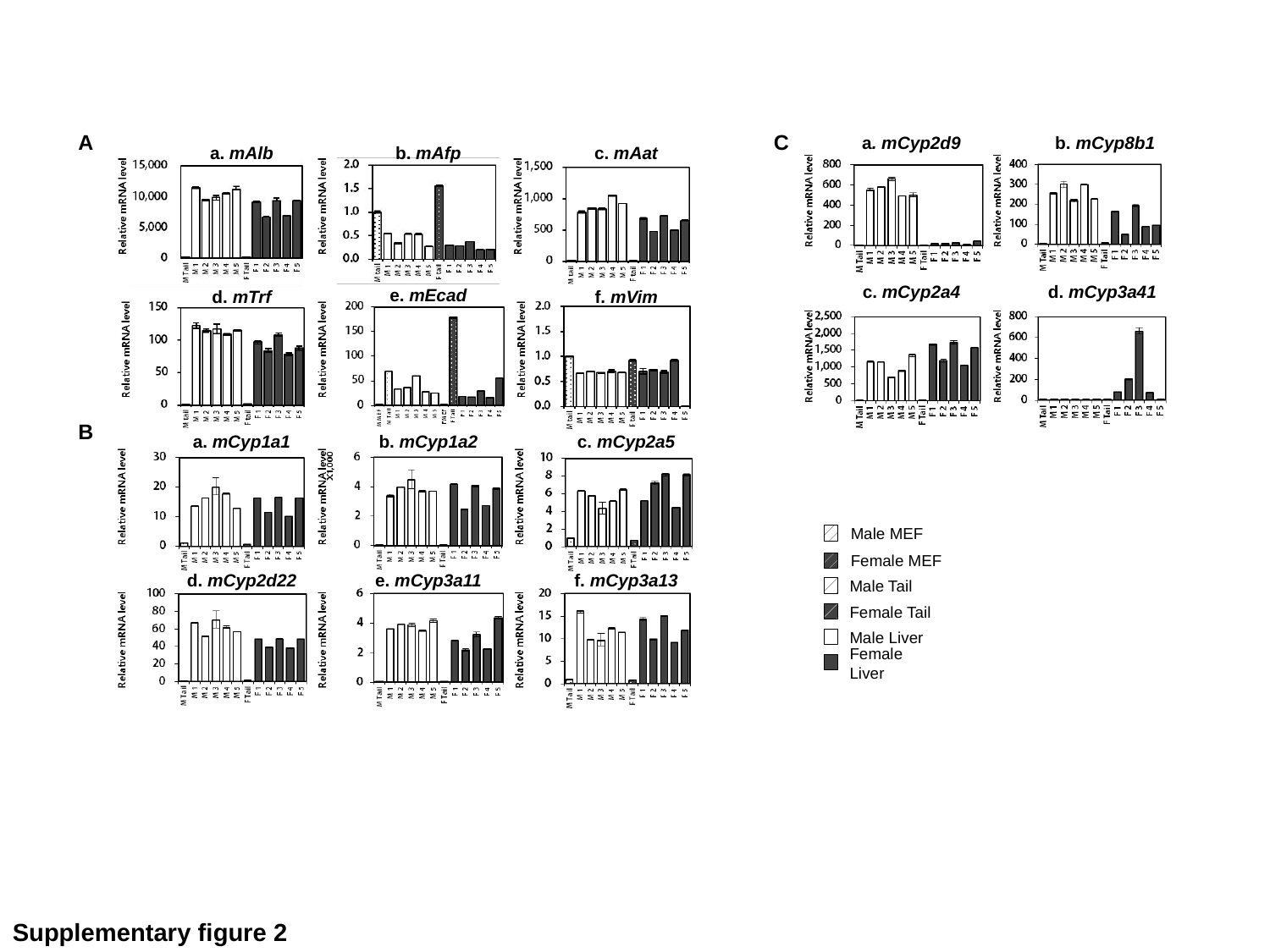

A
C
a. mCyp2d9
b. mCyp8b1
a. mAlb
b. mAfp
c. mAat
c. mCyp2a4
d. mCyp3a41
d. mTrf
e. mEcad
f. mVim
B
a. mCyp1a1
b. mCyp1a2
c. mCyp2a5
Male MEF
Female MEF
Male Tail
Female Tail
Male Liver
Female Liver
d. mCyp2d22
e. mCyp3a11
f. mCyp3a13
Supplementary figure 2

## Slide 3
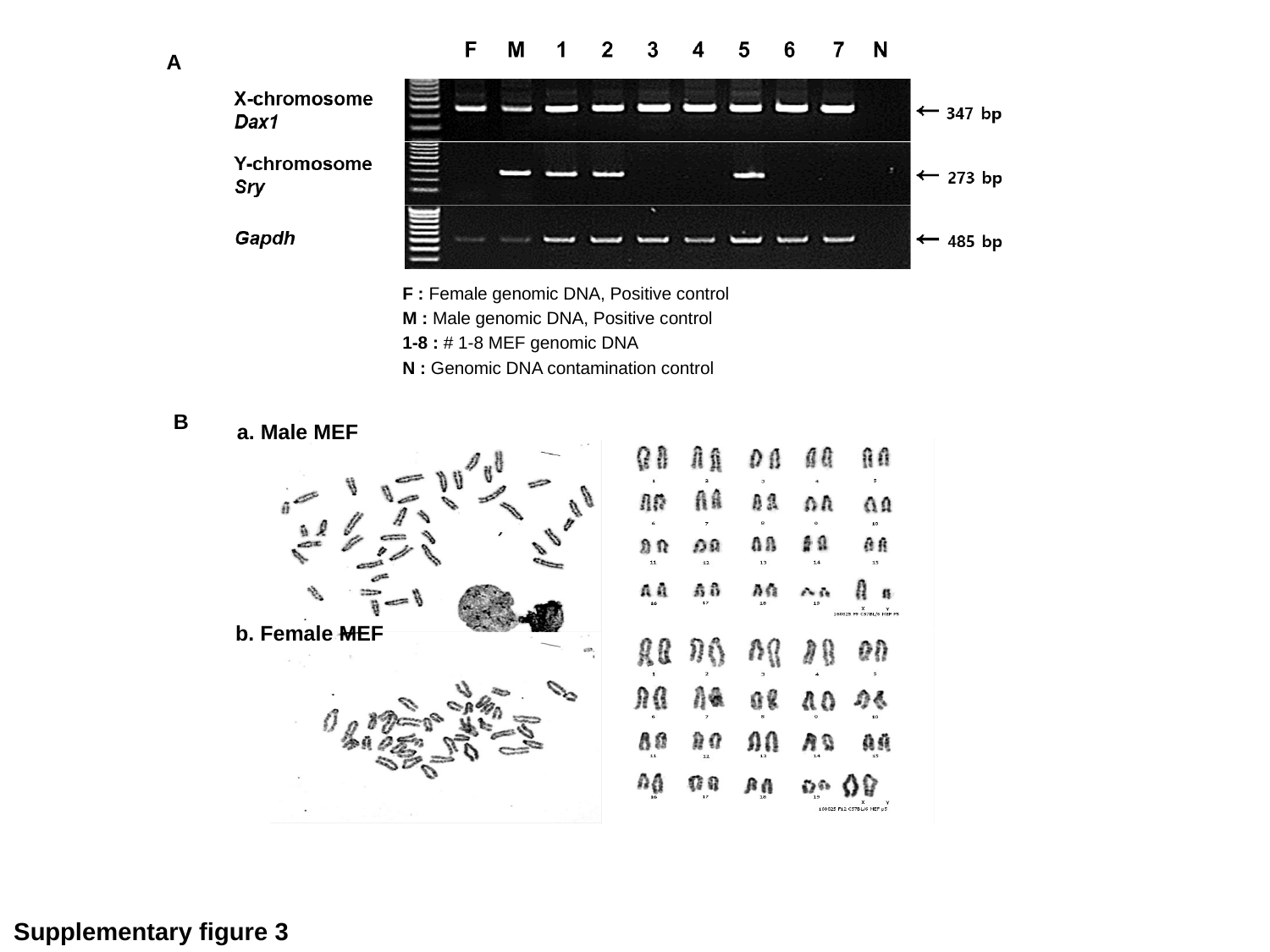

A
F : Female genomic DNA, Positive control
M : Male genomic DNA, Positive control
1-8 : # 1-8 MEF genomic DNA
N : Genomic DNA contamination control
B
a. Male MEF
b. Female MEF
Supplementary figure 3

## Slide 4
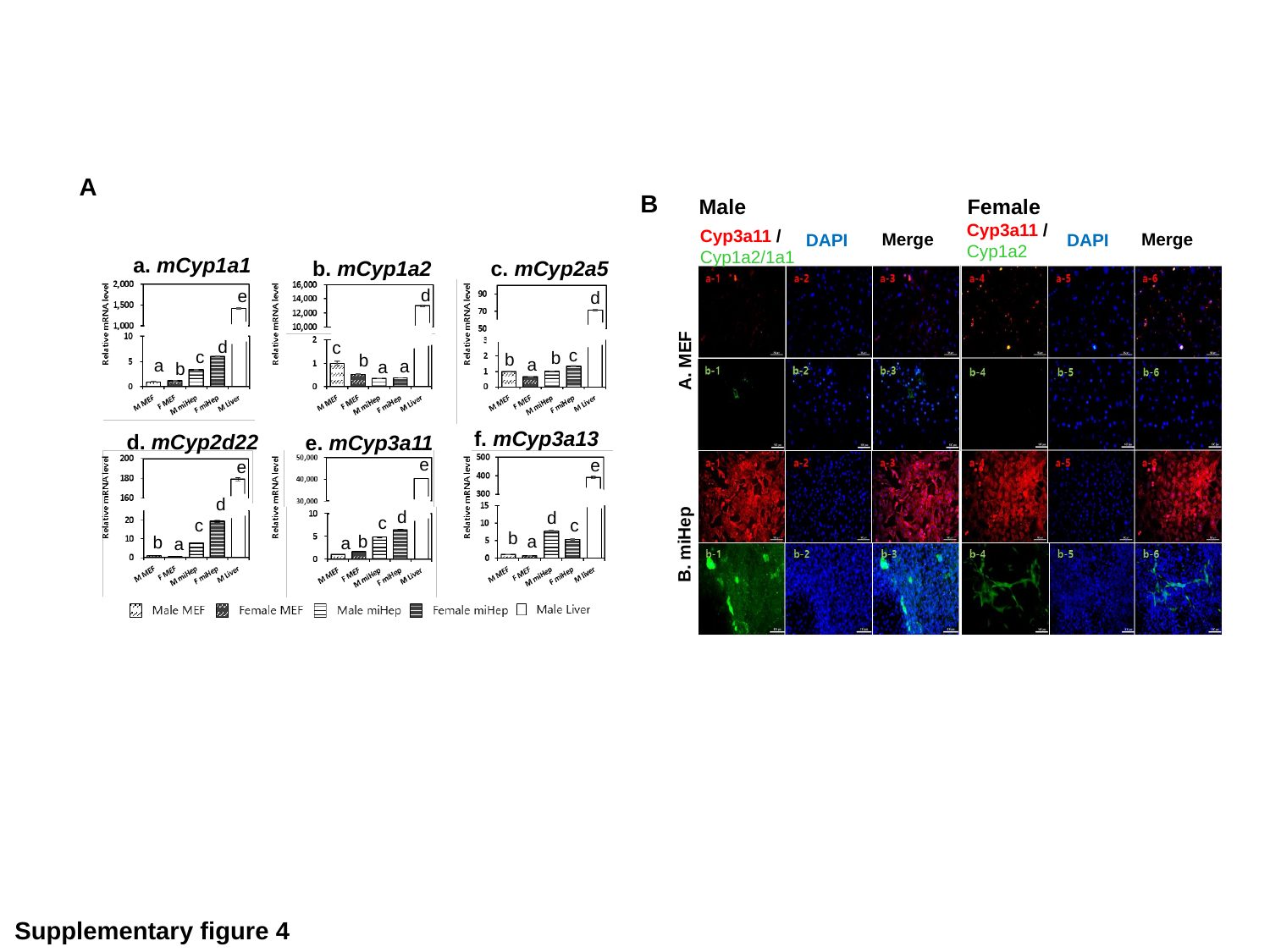

A
Female
Cyp3a11 / Cyp1a2
Male
Cyp3a11 / Cyp1a2/1a1
 Merge
 Merge
DAPI
DAPI
A. MEF
B. miHep
B
a. mCyp1a1
b. mCyp1a2
c. mCyp2a5
d
d
e
d
c
c
b
c
b
b
a
a
a
a
b
f. mCyp3a13
d. mCyp2d22
e. mCyp3a11
e
e
e
d
d
d
c
c
c
b
a
b
b
a
a
Supplementary figure 4

## Slide 5
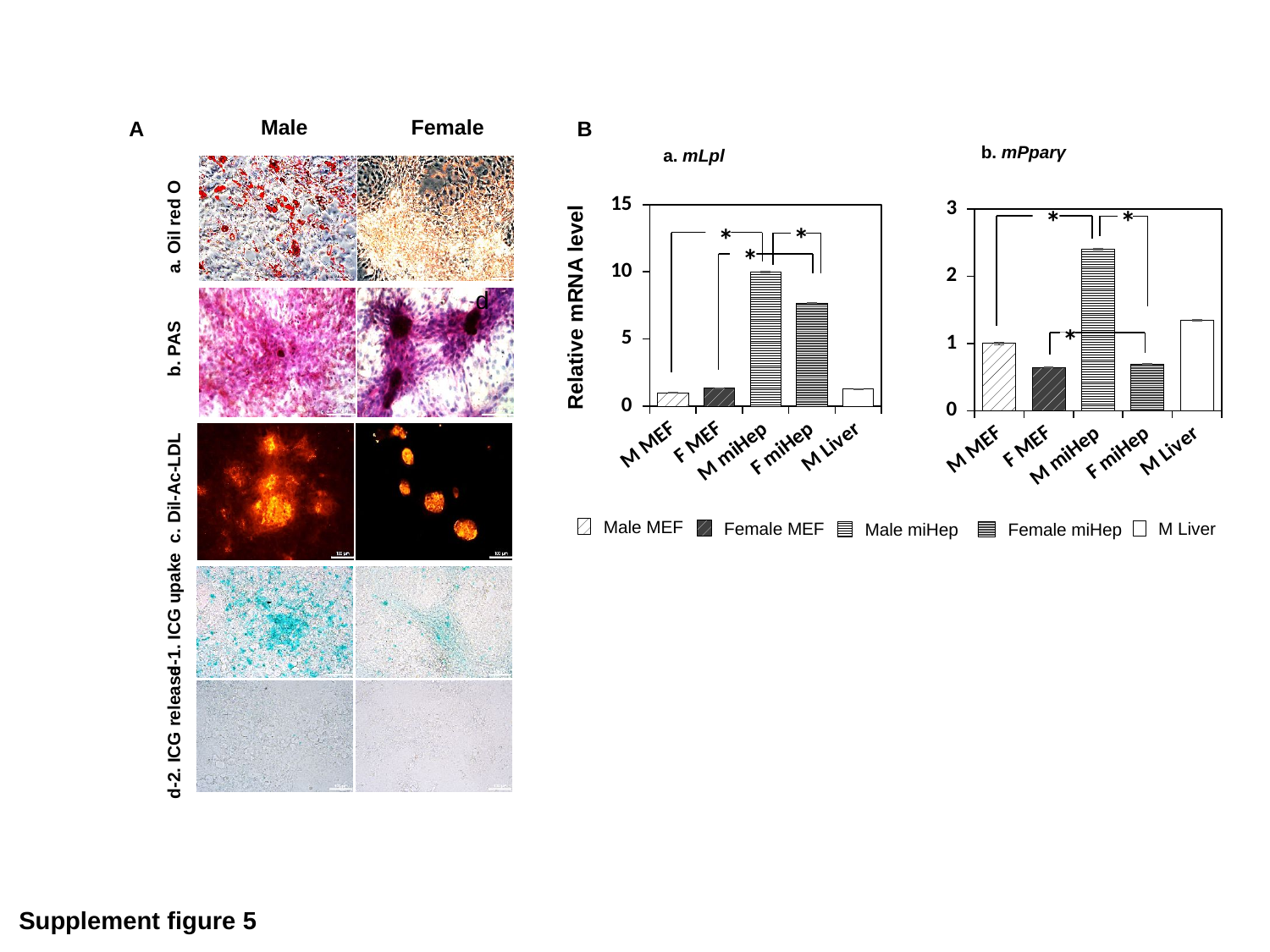

Male
Female
B
A
b. mPparγ
a. mLpl
### Chart
| Category | mLpl |
|---|---|
| M MEF | 1.0 |
| F MEF | 1.343479705237631 |
| M miHep | 9.957980007283805 |
| F miHep | 7.683489418948923 |
| M Liver | 1.2766825374322355 |
### Chart
| Category | mPparg |
|---|---|
| M MEF | 1.0 |
| F MEF | 0.6448642233257271 |
| M miHep | 2.398616613578173 |
| F miHep | 0.6866093901080076 |
| M Liver | 1.3400486682725057 |a. Oil red O
*
*
*
*
*
d
Relative mRNA level
e
b. PAS
*
Relative mRNA level
c
a
c. Dil-Ac-LDL
Male MEF
Female MEF
M Liver
Male miHep
Female miHep
d-1. ICG upake
d-2. ICG release
Supplement figure 5

## Slide 6
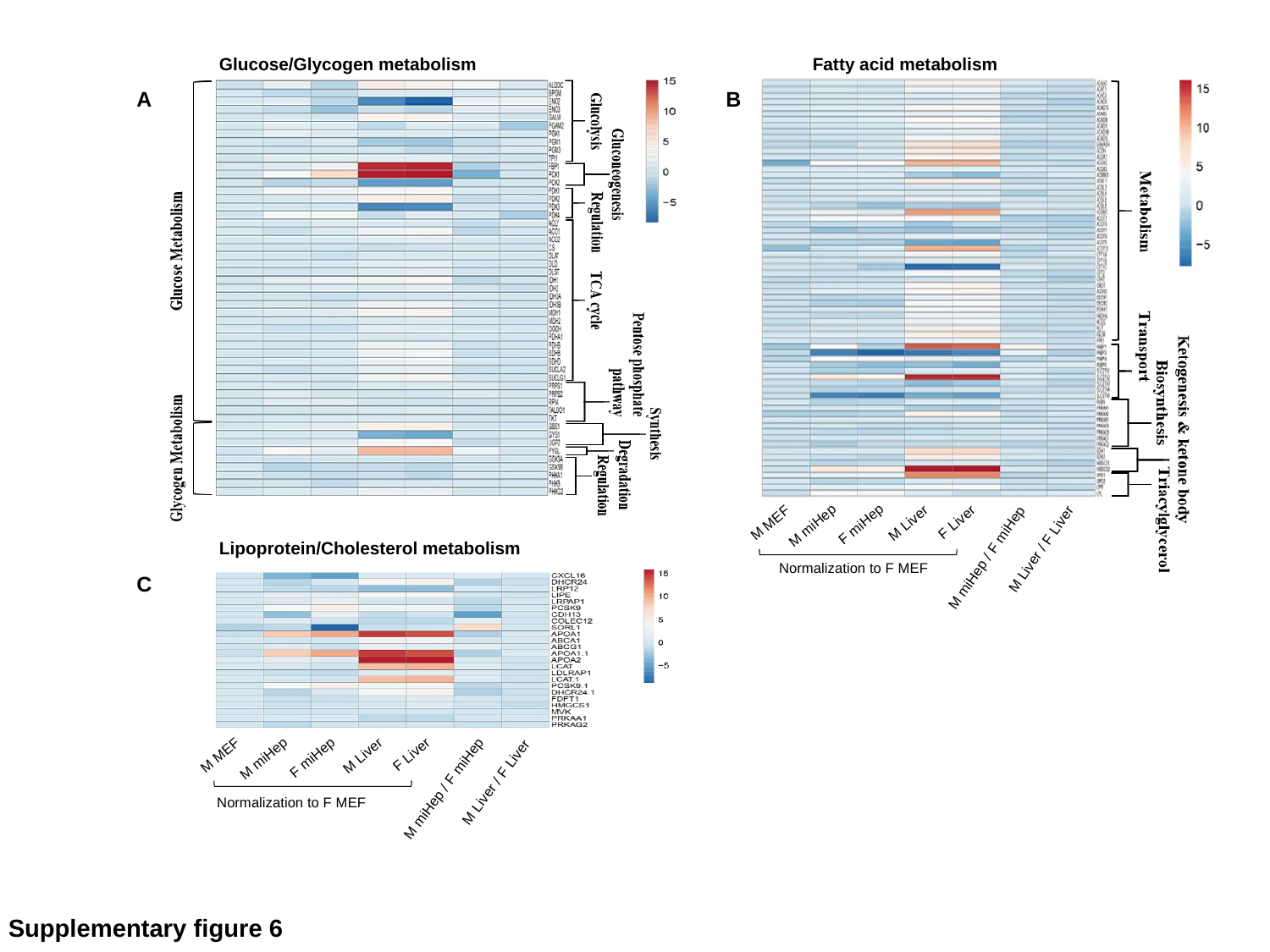

Glucose/Glycogen metabolism
Fatty acid metabolism
A
B
M miHep / F miHep
M Liver / F Liver
M miHep
F miHep
M Liver
F Liver
M MEF
Lipoprotein/Cholesterol metabolism
Normalization to F MEF
C
M miHep / F miHep
M Liver / F Liver
M miHep
F miHep
M Liver
F Liver
M MEF
Normalization to F MEF
Supplementary figure 6

## Slide 7
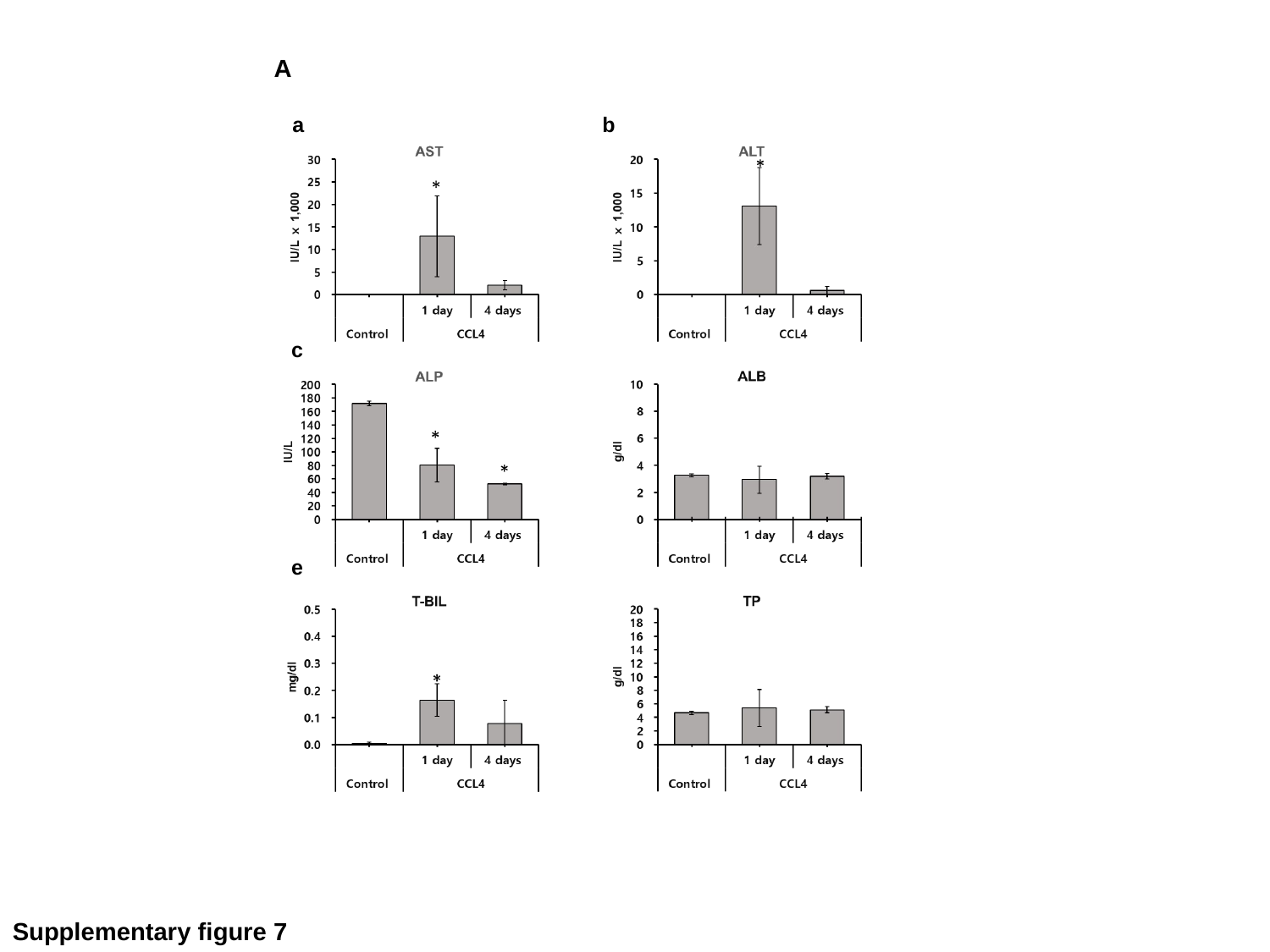

A
a
b
c
e
Supplementary figure 7
