# Supplementary material for: Effect of sex-specific differences on function of induced hepatocyte-like cells generated from male and female mouse embryonic fibroblasts
Source: Stem Cell Res Ther. 2021 Jan 25;12:79. doi: 10.1186/s13287-020-02100-z (PMC7831237; doi:10.1186/s13287-020-02100-z)
Supplement: Supplementary file 2 — Additional file 2: Supplementary Table 1. Primers used for realtime PCR. Supplementary Table 2. Primers used for real-time PCR analysis. Supplementary Table 3. List of antibodies used for immunofluorescence staining. Supplementary Table 4. Ploidy percentage of miHep. [file 13287_2020_2100_MOESM2_ESM.docx]

**Supplementary table 1.** Primers used for real-time PCR

| **Gene** | **Primer (5' → 3')** | | **Product size (bp)** | **Genomic Accession Number** |
| --- | --- | --- | --- | --- |
| *mDax-1* | forward | CATTGACACCAAAGAGTATGCCTATC | 347 | NC_000086.7 |
|  | reverse | GCAGCAGATAATAGAGAGCCAGGTAC |  |  |
| *mSry* | forward | TTGTCTAGAGAGCATGGAGGGCCATGTCAA | 273 | NC_000087 |
|  | reverse | CCACTCCTCTGTGACACTTTAGCCCTCCGA |  |  |
| *mGapdh* | forward | TCGTCCCGTAGACAAAATGGT | 485 | NC_000072.6 |
|  | reverse | CGTTTGCAACATGGCGG |  |  |

**Supplementary table 2.** Primers used for real-time PCR analysis

| **Gene** | **Primer (5' → 3')** | | **Product size (bp)** | **Genomic Accession Number** |
| --- | --- | --- | --- | --- |
| *mAlb* | forward | TGAGATCGCCCATCGGTATAA | 64 | NM_009654.4 |
|  | reverse | GGCAATCAGGACTAGGCCTTT |  |  |
| *mAat* | forward | CCTGCTAAACAGGCGCAGAA | 60 | NM_009243 |
|  | reverse | TCGATGGTCAGCACAGCCTTA |  |  |
| *mAfp* | forward | GCTTCCCTCATCCTCCTGCTA | 61 | NM_007423.4 |
|  | reverse | CAAACTCATTTTCGTGCAATGC |  |  |
| *mTrf* | forward | AGTGTCAGAGCACGAGAATACCAA | 64 | NM_133977.2 |
|  | reverse | CGGAAGGACGGTCTTCATGT |  |  |
| *mEcad* | forward | GAGGAGAACGGTGGTCAAAGAG | 65 | NM_009864.3 |
|  | reverse | AATACACATTGTCCCGGGTATCA |  |  |
| *mVim* | forward | GACCGCTTTGCCAACTACATC | 67 | NM_011701.4 |
|  | reverse | CAGCCAGCAGGATTTTGTTCT |  |  |
| *mCyp1a1* | forward | CGTGAGCAAGGAGGCTAACTATC | 62 | NM_009992 |
|  | reverse | GGCCAACCTCTGCCATCA |  |  |
| *mCyp1a2* | forward | CTGTCCAGGAGCACTACCAAGA | 67 | NM_009993 |
|  | reverse | TTGAACAGGGCACTTGTGATG |  |  |
| *mCyp2a5* | forward | GGACAGGAGGCAGTCAAGGA | 57 | NM_007812 |
|  | reverse | CCGCCCGCTGAATTCC |  |  |
| *mCyp2d22* | forward | GACGACCCTCATCACCAACCT | 60 | NM_019823 |
|  | reverse | GGCTTCTCCCAGACAGTCTCA |  |  |
| *mCyp3a11* | forward | TCACAGACCCAGAGACGATTAAGA | 66 | NM_007818.3 |
|  | reverse | GCCGGTTTGTGAAGACAGAAA |  |  |
| *mCyp3a13* | forward | CCAGTTTACAGATGTGTTGGTGAGA | 66 | NM_007819 |
|  | reverse | ATGCTGGTGGGCTTTCCTT |  |  |
| *mCyp2d9* | forward | TCCCAAGGCTGGCTGACA | 65 | NM_010006.2 |
|  | reverse | CAGGTTATCCAGTATGGCGATGA |  |  |
| *mCyp8b1* | forward | CAGTTTCTGAGGGAGCAAGGA | 68 | NM_010012.3 |
|  | reverse | GCCCAGAGCATCATAAAGTTAAATTT |  |  |
| *mCyp2a4* | forward | CGGACAGGAGACAGTCAAGGA | 60 | NM_009997.2 |
|  | reverse | CCCCGCCCGCTGAA |  |  |
| *mCyp3a41* | forward | CACACTTTCCTTCACCCTGTATTG | 66 | NM_017396.3 |
|  | reverse | TCTTCCTGCAGTTTCTTCTGGAT |  |  |
| *mGapdh* | forward | GAGTGTTTCCTCGTCCCGTAGA | 67 | NM_001289726.1 |
|  | reverse | CGCCCAATACGGCCAAA |  |  |
| *HNF1Α* | forward | CGGAGGAACCGTTTCAAGTG | 58 | NM_000545.6 |
|  | reverse | AGGTTCTTCTGCCTCTCATAGGTTCTTCTGCG |  |  |
| *HNF4Α* | forward | CGCTACTGCAGGCTCAAGAAA | 58 | NM_000457.4 |
|  | reverse | TCTGGACGGCTTCCTTCTTC |  |  |
| *FOXA3* | forward | AGCAGCGCTGGCAGAACT | 62 | NM_004497.2 |
|  | reverse | CACCTTGACGAAGCAGTCGTT |  |  |
| *mHnf1α* | forward | GACCTGACCGAGTTGCCTAATG | 80 | NM_009327.3 |
|  | reverse | GCGAAGTCTTCCCCATCGT |  |  |
| *mHnf4α* | forward | CCAAGAGGTCCATGGTGTTTAAG | 63 | NM_008261.3 |
|  | reverse | GCCGAGGGACGATGTAGTCA |  |  |
| *mFoxa3* | forward | CTTCAACCACCCTTTCTCTATCAAC | 66 | NM_008260.2 |
|  | reverse | TCCAGTTTGGAAGGTGTCGAT |  |  |
| *mLpl* | forward | AAACCCCAGCAAGGCATACA | 62 | NM_008509.2 |
|  | reverse | TCAGGCAGAGCCCTTTCTCA |  |  |
| *mPparγ* | forward | CCCACCAACTTCGGAATCAG | 58 | NM_001127330.2 |
|  | reverse | AATGCGAGTGGTCTTCCATCA |  |  |
| *mGlut2* | forward | CAGCAACTGGGTCTGCAATTT | 62 | NM_031197.2 |
|  | reverse | CAAGGAAGTCCGCAATGTACTG |  |  |

**Supplementary table 3.** List of antibodies used for immunofluorescence staining

| **Gene** | **Antibody** | | **Dilution Factor** | **Time** |
| --- | --- | --- | --- | --- |
| Alb | Primary | Albumin (B-20) IgG (SantaCruz, CA, UAS) | 1:100 | Overnight |
|  | Secondary | Donkey anti-goat IgG-FITC (SantaCrua) | 1:200 | 1 h |
| Aat | Primary | AAT (G-17) IgG (SantaCruz) | 1:100 | Overnight |
|  | Secondary | Donkey anti-goat IgG-FITC (SantaCrua) | 1:200 | 1 h |
| Ecad | Primary | E-cadherin (G-10) (SantaCruz) | 1:100 | Overnight |
|  | Secondary | Goat anti-mouse IgG (H+L), Alexa Flour Plus 594 (Thermofisher Scientific, Barcelona, Spain) | 1:500 | 1 h |
| Vim | Primary | Vimentin monoclonal antibody (RV202) (Thermofisher Scientific) | 1:100 | Overnight |
|  | Secondary | Goat anti-mouse IgG (H+L), Alexa Flour Plus 594 (Thermofisher Scientific) | 1:500 | 1 h |
| Cyp1a2/Cyp1a1 | Primary | Mouse monoclonal antibody IgG1 kappa light chain (SantaCrua) | 1:500 | Overnight |
|  | Secondary | m-IgGκ BP-FITC (SantaCrua) | 1:200 | 1 h |
| Cyp3a11 | Primary | Mouse anti-rat CYP3A1, 3A11 (Millipore) | 1:500 | Overnight |
|  | Secondary | Goat anti-mouse IgG (H+L), Alexa Flour Plus 594 (Thermofisher Scientific) | 1:500 | 1 h |

**Supplementary table 4**. Ploidy percentage of miHep

| Ploidy class | ~ 2c | ~2c-4c | ~ 4c | > ~ 4c |
| --- | --- | --- | --- | --- |
| Male miHep | 36.5± 0.4 | 2.2 ± 0.3 | 39.6 ± 0.9 | 20.8 ± 0.5 |
| Female miHep | 45.7 ± 0.5 | 2.8 ± 1.6 | 44.1 ± 1.8 | 6.6 ± 0.8 |
| Cell cycle | G0/G1 (2c) | S | G2/M (4C) |  |
| Male MEF | 49.6 ± 0.3 | 11.1 ± 2.6 | 38.0 ± 2.0 |  |
| Female MEF | 55.0 ± 0.5 | 12.8 ± 0.3 | 31.2 ± 0.6 |  |
